# Census-independent population mapping in northern Nigeria^☆^

**DOI:** 10.1016/j.rse.2017.09.024

**Published:** 2018-01

**Authors:** Eric M. Weber, Vincent Y. Seaman, Robert N. Stewart, Tomas J. Bird, Andrew J. Tatem, Jacob J. McKee, Budhendra L. Bhaduri, Jessica J. Moehl, Andrew E. Reith

**Affiliations:** a Urban Dynamics Institute, Oak Ridge National Laboratory, Oak Ridge, TN, USA; b Bill & Melinda Gates Foundation, Seattle, WA, USA; c WorldPop, Department of Geography and Environment, University of Southampton, Highfield, Southampton, UK; d Flowminder Foundation, Stockholm, Sweden

**Keywords:** Population, Settlement mapping, Nigeria, Demographics, Polio

## Abstract

Although remote sensing has long been used to aid in the estimation of population, it has usually been in the context of spatial disaggregation of national census data, with the census counts serving both as observational data for specifying models and as constraints on model outputs. Here we present a framework for estimating populations from the bottom up, entirely independently of national census data, a critical need in areas without recent and reliable census data. To make observations of population density, we replace national census data with a microcensus, in which we enumerate population for a sample of small areas within the states of Kano and Kaduna in northern Nigeria. Using supervised texture-based classifiers with very high resolution satellite imagery, we produce a binary map of human settlement at 8-meter resolution across the two states and then a more refined classification consisting of 7 residential types and 1 non-residential type. Using the residential types and a model linking them to the population density observations, we produce population estimates across the two states in a gridded raster format, at approximately 90-meter resolution. We also demonstrate a simulation framework for capturing uncertainty and presenting estimates as prediction intervals for any region of interest of any size and composition within the study region. Used in concert with previously published demographic estimates, our population estimates allowed for predictions of the population under 5 in ten administrative wards that fit strongly with reference data collected during polio vaccination campaigns.

## Highlights

• Human populations are estimated independently of national census data.
• High-resolution settlement mapping is combined with survey data.
• Population density depends on residential settlement pattern.
• Uncertainty quantification is enabled by Monte Carlo simulation.
• Demographic subpopulations can be accurately estimated.

## 1 Introduction

Current and spatially precise population estimates are a critical data input for efforts in governance, planning, and public health. Without an accurate count or estimate of the population denominator for an area, rates describing demographic compositions, births and deaths, disease incidence, health intervention coverage, technology penetration, service accessibility and voting turnout, for instance, are both difficult to measure and of limited value in future planning. More than one-third of the indicators established to measure progress on the United Nations (UN) Sustainable Development Goals (SDGs) (United Nations, 2016) are defined in terms of total population or a specific demographic subpopulation, despite the fact that the capacity to measure these denominators varies greatly from country to country, especially when data are needed for small areas, rather than at national or provincial levels.

One example of the critical need to ascertain populations for small areas can be found in the work of the Global Polio Eradication Initiative (GPEI) in Nigeria, which conducts regular vaccination campaigns with the aim of vaccinating every child under the age of five. Despite a host of innovative interventions in recent years (Vaz et al., 2016), the polio eradication effort in Nigeria has been hampered by areas of insecurity and a lack of access to all communities and children. The limited access, along with the inadequacy of available geodemographic data, make the accurate assessment of vaccination coverage a challenge, compromising the GPEI's ability to assess the effectiveness and efficacy of the vaccination campaigns (Barau et al., 2014). Even in the ideal case, when supplies, logistics, and freedom to operate allow access to all children in all areas, not knowing where all vaccine-eligible children reside can lead to children being missed by the campaigns. Similarly, the effectiveness of the routine immunization services provided by local health districts cannot be measured without an accurate target population denominator.

For locating and quantifying the number of vaccine-eligible children, national census data have limitations. The last national census in Nigeria occurred in 2006 and provided counts of the total population as well as the populations by sex and 5-year age groups at the level of the Local Government Area (LGA). This level of aggregation did not allow determination of the population of individual settlements within LGAs, a problem the National Population Commission acknowledged and attributed to the lack of authoritative lists and maps of localities (National Population Commission, 2009). Now, a decade removed from the census, ascertaining the population of large or small areas in Nigeria is even more problematic, as the differential growth rates among LGAs over that time is not accounted for in tabular projections using constant growth rates. The case of Nigeria is far from unique, and it is representative of the challenges faced by governments and NGOs attempting to implement ambitious programs in countries where the availability of detailed geographic and demographic data is inadequate (Tatem and Linard, 2011).

Although settings without recent and reliable census data are common, most research in spatially precise population estimation relies on national census data for observations of population counts. A common approach is to estimate a population density for each class of land cover or land use, whether by regressing the census populations on the areas of the different land classes (Fisher and Langford, 1995, Goodchild et al., 1993, Langford et al., 1991, Yuan et al., 1997) or by compiling an empirical sample for each class by identifying enumeration units that are completely (or mostly) covered by a single class (Mennis, 2003, Mennis and Hultgren, 2006). The census data can also be used to constrain estimates so that sums are preserved within the enumeration units. Whether this constraint is imposed depends on whether the goal of the estimation is a real interpolation of the census counts or predictions outside of the context of model training, whether for different regions or dates (Wu et al., 2005). Further refinements of census-based methods include incorporation of additional ancillary data in combination with land cover (Dobson et al., 2000, Stevens et al., 2015) and the application of alternative spatial denominators (other than area), such as building volume (Sridharan and Qiu, 2013), street lengths (Reibel and Bufalino, 2005), or residential address points (Zandbergen, 2011).

While the land classifications used in some early population estimation work were hand-drawn and guided by “controlled guesswork” (Wright, 1936), most modern techniques use data derived via remote sensing. Although a variety of remote sensing data and methods have been applied to population estimation problems, the increasing availability of high-resolution optical and radar imagery has contributed to a gradual trend, recognized at least as early as 2004 (Tatem and Hay, 2004), toward window-based textural classifications, which have been shown to be well suited for identifying and characterizing the complex structures of human settlements (Cheriyadat et al., 2010, Martino et al., 2003, Pesaresi, 2000). (Unless otherwise noted, our discussion of resolution throughout the text refers to spatial resolution.) In order to deploy these principles at regional and global scales, scalable workflows have been developed within computational platforms such as the Global Human Settlement (GHS) framework at the Joint Research Centre (JRC) of the European Commission (Pesaresi et al., 2013), the Settlement Mapper Tool (SMT) platform developed at Oak Ridge National Laboratory (ORNL) (Cheriyadat et al., 2007, Patlolla et al., 2012), and the German Aerospace Center (DLR)'s Urban Footprint Processor (UFP) (Esch et al., 2013). The highest-resolution settlement layer with global coverage from these platforms is currently DLR's Global Urban Footprint (GUF), which can be licensed at 12-meter resolution for scientific use. Higher resolutions of 10 m (Florczyk et al., 2016) and 8 m (Patlolla et al., 2012), respectively, have been demonstrated with the GHS and SMT platforms, but global coverages do not exist at these resolutions.

Although most population estimation relies on census data, there are a handful of relevant examples of census-independent (“bottom-up”) approaches to mapping residential populations in data-poor environments. In one approach (Checchi et al., 2013), density estimates derived from literature and internet sources were used in conjunction with manual counts of structures from satellite imagery to estimate counts of displaced persons in eleven sites (a mixture of camps and urban neighborhoods) in Asia and Africa, and the largest estimation errors were seen where the density reports were scarce or unreliable, and/or where individual structures were difficult to discern from imagery. Another study (Hillson et al., 2014) used field surveys in Bo, Sierra Leone, to gather population observations and manual image interpretation to count buildings and measure their rooftops. An occupancy-based model (people per structure) was found to be more accurate than a rooftop area-based model, but the authors stressed the importance of practical considerations when choosing a density denominator. A third study (Stewart et al., 2016) estimated daytime and nighttime population using population density models derived from literature and internet sources and linked to specific facility types. Again, building footprints and classifications were identified manually from satellite and street-level imagery.

In this paper, we tackle the problem of unreliable and outdated census population counts through a bottom-up population mapping approach that couples semi-automated high-resolution settlement mapping with microcensus surveys, which are enumerations for sample zones within the settlement area, to estimate residential populations without relying on national census data. Our primary focus is on estimating the total residential population with high spatial precision, which can then serve as the denominator for estimating subpopulations when used in concert with known or estimated demographic, socioeconomic, or epidemiological rates (or, conversely, for estimating such rates in concert with observations of the numerators). We demonstrate subpopulation estimation by estimating the population of children under 5, a key demographic group for many health and development applications, including polio eradication. But this is just one possible application; the core of our approach has general applicability for any initiative aiming to accurately locate human settlements and estimate (sub-) populations in regions where census data are outdated or spatially imprecise.

## 2 Methods

### 2.1 Overview

Our approach to estimating residential population counts relies on three major components: a binary spatial layer of human-inhabited areas (the settlement layer), a categorical spatial layer of residential settlement types (the residential type layer), and a model of population density. The settlement layer and residential type layer are generated through remote sensing methods, while the population density model is specified using survey data from a microcensus.

**Fig. 1:**
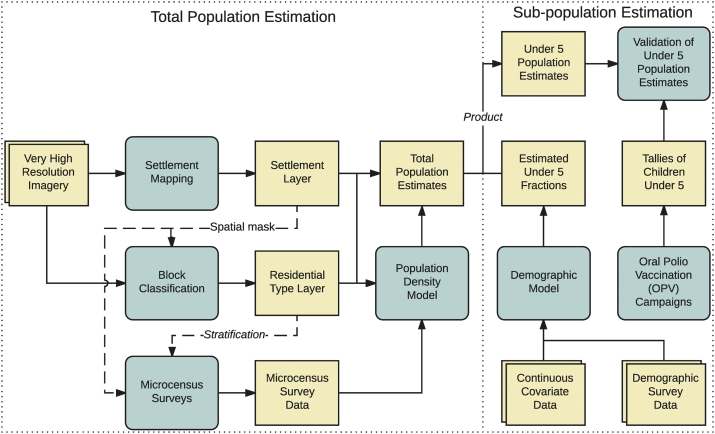
Overview of the population modeling approach. Rounded blue boxes indicate a major data collection, processing, or modeling component. Squared tan boxes indicate data, either collected or generated within this workflow. All data items have a spatial component. (For interpretation of the references to color in this figure legend, the reader is referred to the web version of this article.)

To demonstrate and validate an approach to applying the population estimates toward the estimation of a subpopulation, we introduce a fourth component, a set of previously published demographic estimates (Alegana et al., 2015). We use the published estimates of the under-5 fraction of the population in conjunction with our population estimates to derive estimates of under-5 population counts for ten wards in Kano state, for which independent validation data are available. A graphical outline of the overall approach is shown in Fig. 1.

From the top-down modeling literature, we borrow the concept of estimating populations using land classes, but we adapt it to a census-independent setting. Our approach resembles that of Mennis and Hultgren (2006), but rather than selecting representative census enumeration units as our sample, we instead employ field observations (microcensus surveys) for density estimation, and rather than use relatively coarse land cover data, we map and classify settlements at a very high resolution using recent imagery. Our use of surveys recalls the bottom-up approach used by Hillson et al. (2014), but rather than manually delineating rooftops, we employ automated settlement mapping, which more readily enables scaling across large areas. The density denominator we use in our modeling is *area of human settlement*, which is the denominator we can most readily and accurately measure across the entire study region.

We assess the accuracy of our approach with two analyses. The first is the validation of our under-5 population estimates using enumerations collected as part of polio vaccination campaigns, and the second is a comparison of our model with 2006 census counts and constant-growth estimates derived therefrom, which we enable by repeating our bottom-up mapping approach with 2006-vintage imagery for the metropolitan area of Kano.

### 2.2 Study region

The study region consists of the states of Kano and Kaduna, in the center of northern Nigeria. Three large cities, Kano, Kaduna, and Zaria, lie within the study region, surrounded by many rural settlements of various sizes. At the time this work was initiated (early 2014), this region was still experiencing polio cases and was considered to be the key battleground in the fight for eradication in Nigeria. The city of Kano (the capital of Kano state) is the largest in northern Nigeria and serves as a hub for resources and infrastructure related to a variety of humanitarian efforts in the region, including extensive GIS database development to support the polio effort (Barau et al., 2014).

### 2.3 Settlement mapping

SMT is a machine learning system for extracting settlement areas from very high-resolution imagery (Cheriyadat et al., 2007, Patlolla et al., 2012). This system classifies 16 × 16-pixel blocks into settled and non-settled regions using a support vector machine (SVM) classifier that is trained on various low-level contextual image features. The overall system is built on the principle that the spatial arrangements of structural attributes of the built environment (building edges, building corners, linear infrastructure) are better indicators of settlement than are per-pixel spectral measurements. Therefore, features that represent textural and structural attributes are prioritized. These include Histogram of Oriented Gradients (HOG) (Dalal and Triggs, 2005), Gray Level Co-occurrence Matrix (GLCM) (Cheriyadat et al., 2007, Haralick et al., 1973, Martino et al., 2003, Pesaresi et al., 2008), textons (Malik et al., 2001), and Dense Scale Invariant Feature Transform (Dense SIFT) (Lowe, 2004). For additional spatial context, the features are calculated at multiple scales surrounding each pixel block. SMT exploits the graphics processing unit (GPU) cores of high performance machines to rapidly extract settlements (Patlolla et al., 2012).

We obtained 3-band WorldView 2 imagery for all of Kano state from DigitalGlobe and 4-band Pléiades 1A and 1B imagery for all of Kaduna from Airbus. Both sets of imagery were pan-sharpened at 0.5 m spatial resolution. In areas of overlapping imagery, the best imagery was selected based on date and cloud cover; we selected 37 image strips in Kano and 97 strips in Kaduna. For 90.3% of the area of the two states, the imagery dates were from 2013 (68.7%) or 2014 (21.7%). The remaining 9.7% of the area required imagery from 2010 to 2012 due to cloud cover in the more recent imagery.

For each image strip, analysts with college and/or graduate-level training in GIS and remote sensing performed heads-up digitizing of representative settled and non-settled areas within the SMT interface to train an SVM model. After training, the model was applied to the remainder of the image strip. Each model output was reviewed and approved by a senior analyst, and the raster output, at a resolution of 8 m (16 times the 0.5 m image resolution), was converted to a polygon vector format, which was then given a more detailed review by a third analyst, who edited polygons as needed to correct obvious commission and omission errors. The most common commission errors occur at locations where linear features and sharp gradients in reflectance are confused for buildings, such as along sandy riverbanks and rural highways. Omission errors usually occur in very small settlements, often in settlements with round huts with thatched rooftops, which do not exhibit the reflectance gradients or the straight lines typical of most other buildings in the region. Finally, a senior analyst reviewed and approved each edited output.

The edited settlement polygons were converted back to raster format at a resolution of 0.25 arc-seconds (≈ 7.7 m at the equator), which approximates the 8-m resolution of the original results but is optimal for aggregating neatly to the target resolution of the gridded population layer, which is 3 arc-seconds (≈ 93 m). The 3 arc-second resolution in turn aggregates neatly to the 30 arc-second (≈ 930 m) resolution of several well-known global and regional gridded population datasets, such as LandScan (Dobson et al., 2000), WorldPop (Tatem, 2017), GRUMP (Center for International Earth Science Information Network et al., 2011), and GPW (Doxsey-Whitfield et al., 2015).

### 2.4 Block classification

The classification of settlement types was accomplished through a combination of supervised image segmentation and manual correction of errors and identification of non-residential land uses, all within the framework of a topological set of polygon “blocks”. The blocks were constructed from a selection of line features from OpenStreetMap data, including roads and hydrographic features. The blocks provide a useful preliminary structure, because boundaries between different residential types (and between residential and non-residential land uses) often follow natural or infrastructural features (e.g., streams or roads). During manual review and editing, some blocks were subdivided as needed when multiple settlement types can be seen within a single block. Subdivision boundaries were drawn to follow visible linear features whenever possible.

Each block (or sub-block, if split) was assigned a letter code corresponding to its use type. These codes include six urban residential types (*A*–*F*), one rural residential type (*M*), and a non-residential type (*Z*). As in all supervised classification procedures, the target typology had to be developed a priori, which we accomplished by inspecting imagery across the study region and identifying visually distinct settlement patterns. Representative images of the urban residential types are shown in Fig. 3. Supervised factorization-based texture segmentation (FSEG) (Yuan et al., 2015) was used to develop an initial layer of settlement types for each large urban settlement in each state, and this process was followed by a post-processing and review workflow.

The result of the supervised segmentation process was a per-pixel classification, which was then summarized at the block level, in such a way that the fraction of the settlement in each block corresponding to each settlement type was recorded. For example, a block may be summarized with the attributes, (*A* = 0.92, *D* = 0.03, *F* = 0.05). Each block was automatically assigned an initial type corresponding to the largest portion (which is *A* in this hypothetical example). Manual review and editing of the results was then undertaken, which prioritized the most heterogeneous blocks, as those blocks are most likely to be misclassified and/or require subdividing into sub-blocks. Blocks above a threshold of 90% homogenous were typically left alone unless an analyst could clearly identify a misclassification error.

During manual review of the segmentation results, additional ancillary data was used to identify non-residential land uses, especially where they only occupy a portion of a block. Sub-blocks of the non-residential type *Z* were carved from the blocks to represent these non-residential uses. The ancillary information used to guide this process included spatial point and polygon data representing schools, medical facilities, mosques, markets, etc., from the Vaccination Tracking System (VTS) database (Barau et al., 2014), the Nigeria MDG Information System (NMIS) (Center for Sustainable Development, 2014), and volunteered geographic information (VGI) sources: OpenStreetMap, Google Map Maker, and Wikimapia.

### 2.5 Microcensus surveys

Microcensus surveys were conducted by a local NGO (eHealth Africa) in Kano and Kaduna to enumerate residents in representative locations, in order to inform the population density model. Each microcensus location was defined by a sample point and a corresponding microcensus enumeration zone (MEZ), which was manually delineated around each sample point. The zones were drawn to follow roads and other logical features, so that the boundaries would be clear to the surveyors. Each MEZ covered approximately 25–50 residences. Every building in each MEZ was visited and a population count for each building (or tightly linked complex of buildings) was recorded in a database. The total cost of data collection, including surveyors' time, transportation, and data cleaning, was approximately $600 per MEZ. A population density was calculated for each MEZ by dividing the sum of the per-building counts by the area of the spatial intersection of the MEZ and the settlement layer.

Two datasets of population density observations were collected as part of the microcensus. Dataset 1 (DS1) consists of population densities observed in the first round of the microcensus, in which a random sample of 100 locations was taken within the entire settlement layer, without any stratification across the different residential types (though the locations were stratified across the two states—50 in Kano and 50 in Kaduna). Because there was no stratification by type, DS1 consists mostly of rural density observations, since the rural type (*M*) is by far the most prevalent type in terms of area. In order to increase sample sizes for urban types, a second dataset (DS2) was collected in a second round of the microcensus, in which sample locations were stratified across the portions of the settlement layer corresponding to the different urban residential types. Twelve locations were selected from each type (six in each state). Sampled locations were discarded if they fell within 500 m of a location already in the sample, which eliminated the possibility of overlapping MEZ polygons. This condition substantially reduced the sample size of type *E*, which covers so little area as to make it impractical to identify 12 locations that are all at least 500 m from every other point. One sample location fell within a block originally identified as type *F*, but which was later (after sampling was complete) found to have been misclassified. The block was corrected to type *D*, resulting in thirteen sample locations for type *D* and eleven sample locations for type *F*. The two datasets are summarized in Table 1.

**Table 1:** Summary of microcensus datasets. Population densities are in persons per hectare.

	Overall	Residential type						
		A	B	C	D	E	F	M
DS1								
Number of sample locations	100	1	15	3	6	0	3	72
Mean of sample areas (ha)	3.16	0.82	2.74	2.89	4.50	–	8.53	2.95
Total area sampled (ha)	315.74	0.82	41.13	8.67	26.98	0.00	25.59	212.54
Mean population density	245.2	843.0	281.7	249.3	136.0	–	58.0	246.0

DS2								
Number of sample locations	67	12	12	12	13	7	11	0
Mean of sample areas (ha)	2.55	1.38	1.64	2.42	3.08	1.22	5.18	–
Total area sampled (ha)	170.73	16.51	19.69	29.00	40.09	8.51	56.94	0.00
Mean population density	321.6	617.3	405.3	307.5	159.7	401.3	63.7	–

Combined (DS1 + DS2)								
Number of sample locations	167	13	27	15	19	7	14	72
Mean of sample areas (ha)	2.91	1.33	2.25	2.51	3.53	1.22	5.89	2.95
Total area sampled (ha)	486.47	17.33	60.82	37.67	67.07	8.51	82.53	212.54
Mean population density	275.8	634.6	336.6	295.9	152.2	401.3	62.5	246.0

### 2.6 Population density modeling

Residential population density, in terms of residents per unit of settlement area, can be understood as the multiplicative product of several variables, including the spacing of buildings, building height, and the number of residents per floor area within households. These multiplicative effects result in a positively skewed, log-normal distribution of densities (Limpert et al., 2001). The distributions of values by type (across both DS1 and DS2) are shown as a boxplot in Fig. 4.

In this and subsequent sections, a random variable will be denoted with a capital letter (e.g., *D*) and, if needed, a categorical or object indicator as a subscript (e.g., *D*_*t*_), while a vector of observed realizations of a random variable will be denoted with a corresponding lowercase letter, possibly subscripted with an index (e.g., *d*_*i*_), a categorical indicator (e.g., *d*_*t*_), or both (e.g., *d*_*ti*_). A vector of simulated realizations of a random variable will be denoted with a lowercase letter with a prime symbol (e.g., *d*^′^).

The distinct sampling approaches (non-stratified and stratified) represented by DS1 and DS2 allowed for two modeling approaches to be carried out and compared. The first, Model 1, represents a hypothetical scenario in which settlement mapping results are available, but a residential classification is not. Therefore, all density observations from DS1, regardless of type, were pooled together and described by a single log-normally distributed random variable *D*:

(1) $$D\sim \mathrm{Lognormal}\left(\mu ,\sigma^{2}\right)$$

where *μ* and *σ* are the mean and standard deviation of the natural logarithm of *D* and were estimated from the DS1 density observations.

We do not propose Model 1 as the most appropriate model given the data we have collected, but instead specify it only as a control with which to contrast our proposed model, Model 2, which does take the residential types into account. Fig. 4 shows that the population densities are different across types, in terms of means but also in terms of variances, and all types exhibit clear positive skewness, except type *E*. Because of the clear differences among the means and variances, Model 2 consists of 8 sub-models—one for each residential type, and one for the non-residential type. For each residential type, the combined density observations from DS1 and DS2 are described by a type-specific log-normal distribution, while the densities of non-residential locations (type *Z*) are fixed at zero:

(2) $$\begin{array}{l} D_{t}\sim \mathrm{Lognormal}\left(\mu_{t},{\sigma^{2}}_{t}\right),\;\;\mathrm{if}\;t\in \left\{A,B,C,D,E,F,M\right\}\\ D_{t}=0,\;\mathrm{if}\;t=Z \end{array}$$

### 2.7 Gridded population estimates

To make point estimates of population counts, a raster layer of population density was produced by joining the relevant mean estimate from Model 2 to each classified block polygon and converting the polygons to a raster format. Population count estimates were first computed at a resolution of 0.25 arc-second (≈ 7.7 m), and the results were then aggregated to the final 3-arc-second (≈ 93 m) resolution. The population count for each 0.25-arc-second cell is the product of three input rasters: the density raster, representing the modeled population density estimate in residents per hectare (residents per 10,000 m^2^) for every location; the settlement raster, representing presence or absence of settlement (1 or 0); and an area raster, representing the area of each cell, in hectares. The estimated population count in each 3 arc-second cell is the sum (rounded to yield an integer) of the population estimates of the cell's 144 constituent 0.25 arc-second cells.

### 2.8 Population prediction intervals

To quantify uncertainty for zonal analyses (e.g., how many residents are in a particular city ward), we can specify a prediction interval of the form, [*lower*, *upper*], for any region of interest (ROI), such that:

(3) $$\mathrm{Prob}\left(C_{\mathrm{ROI}}<\mathrm{lower}\right)=\mathrm{Prob}\left(C_{\mathrm{ROI}}>\mathrm{upper}\right)=\frac{1-p}{2}$$

where *C*_*ROI*_ represents the residential population count in the region of interest and the confidence level is given by *p*. Alternatively, this interval can be divided by the area of the ROI to yield an interval in density terms at the same confidence level.

To generate an interval for an ROI, we employ a Monte Carlo simulation method. We begin by defining a reference scale for each type. For each residential type, we have a set of observed populations measured over roughly equal settlement areas, and the mean of the measurement areas serves as that type's reference area, ɸ (in hectares). We calculate a new reference population density for each observation by multiplying the density by ɸ, in order to express each density in terms of the reference area. The distribution of these reference densities has the same shape as the corresponding distribution from Eq. (2), but the parameters are on a different scale due to the change in the area denominator. Therefore, we have

(4) $$D_{t\phi }\sim \mathrm{Lognormal}\left({µ}_{t\phi }{\sigma^{2}}_{t\phi }\right)$$

where *D*_*tɸ*_ is a random variable describing the distribution of population densities of type *t* for areas of size ɸ, in terms of residents per ɸ.

Zonal queries of the data may occur for any aggregate target area, *area*_*ROI*_ ∈ {*k*ɸ | *k* ≥ 1}. Determining prediction intervals requires evaluating the quantiles of the distribution of the random variable, *C*_*ROI*_, which describes the possible residential population counts for regions having the size and type-composition of the ROI. We treat the problem as one of summing independent and identically distributed (i.i.d.) random variables, and we approximate the solution via simulation. In the simplest case of an ROI of only one type, which is *k* times the size of ɸ (and *k* is an integer), this involves summing *k* instances of *D*_*t*ɸ_, to approximate the *k*-fold self-convolution of the distribution of *D*_*t*ɸ_. Specifically, we make *k* random draws from the *D*_*t*ɸ_ distribution to arrive at one realization of the total population in the ROI. (To include parameter uncertainty, *D*_*t*ɸ_ is randomly altered for each draw, per the standard errors of its parameter estimates.) We generate 10,000 such realizations, resulting in a simulated vector, *c*_*ROI*_^′^, which represents population counts for 10,000 hypothetical zones of the same size (*k*ɸ) as the ROI. We can then evaluate the quantiles at [0.05, 0.95] of *c*_*ROI*_^′^ to yield a prediction interval for *p* = 0.9. (If population density is desired, dividing each value in *c*_*ROI*_^′^ by *k*ɸ gives a vector *d*_*ROI*_^′^ of simulated densities.)

In practice, ROIs will often contain more than one residential type, and *k* will almost never be an integer. Therefore, simulations of actual ROIs must often include draws from multiple distributions and must handle the non-integer component of *k*. For example, the ward of Rijiyar Lemo (in Fagge LGA, Kano state, Nigeria) consists of portions of the residential types *B*, *D*, *E*, and *M*, each of which has a different reference distribution and a different non-integer *k*. The area of the ward covered by each type, along with the type's reference area, ɸ_*t*_, and the applicable area factor, *k*, are shown in Table 2.

**Table 2:** Area by type in Rijiyar Lemo ward, Fagge LGA, Kano state, Nigeria.

Type	Area (ha)	ɸ (ha)	Area factor (*k*)
B	37.826	2.25	16.81
D	2.899	3.53	0.82
E	40.890	1.22	33.52
M	1.0745	2.95	0.36

In a multi-type case, each simulation begins with one realization of each type's portion of the ROI. For example, for type *B* in Rijiyar Lemo, with an area factor of 16.81, seventeen draws are simulated (the seventeenth draw represents an area smaller than ɸ and is therefore scaled by multiplying by 0.81). One realization of the entire ward is the sum of the realizations of the types. Again, we repeat this 10,000 times to generate a simulated vector of possible population counts, the quantiles of which can be used to define prediction intervals for the true population of the ward.

### 2.9 Estimating and validating sub-populations

One of the primary uses of estimates of the total population is as a denominator for estimates of subpopulations, about which we may know rates (e.g., percent female, percent impoverished) but not totals. Here we demonstrate one such application, the estimation of the population under 5 years of age, which is of interest for a variety of public health applications, including polio eradication. Rates come from a set of previously published demographic estimates (Alegana et al., 2015), which were generated by a model that used freely available household survey data. The estimates are gridded and have a resolution of 1 km^2^.

We generated estimates of the under-5 population in ten wards in Kano. These wards were chosen due to the availability of an independent dataset that could be used for validation. The validation dataset is the result of a pilot program to implement an electronic tally (eTally) to supplement the paper tallies collected as part of the oral polio vaccination (OPV) campaigns in northern Nigeria. The campaigns are designed to ensure all children under five years of age are vaccinated, and are planned and implemented at the ward level, with teams visiting every household over a four-day period. Traditionally, the teams manually record on a paper tally sheet the number of children under 5 years of age residing at each house and the number of children vaccinated. At the end of the campaign, the “administrative coverage” is calculated (the fraction of the target population vaccinated) to assess quality and effectiveness of the campaigns. Since July of 2015, the eTally has been conducted in selected areas to provide more timely and accurate data for vaccination tracking. An additional supervisor accompanies the vaccination teams and collects the tally sheet data using a custom application on a GPS-enabled Android phone. The eTally data are uploaded each day to the VTS website (Barau et al., 2014). The eTally was expanded in late 2015 and early 2016, ultimately covering 162 Wards in 32 states.

To check the reliability of the eTally data, the activity was repeated in two Wards in Kano, Dugurawa and Rijiyar Lemo, in successive campaigns (July–Sep). The July campaign data were collected by locally-hired supervisors, while the data for September were collected by specially-trained staff of eHealth Africa, who also managed the training and logistics for the project. The results (total number of children under 5 years of age) for the August–September campaigns were nearly identical: 1030 vs 1015 children, respectively, in Dugurawa, and 6309 vs 6376 in Rijiyar Lemo.

The eTally pilot included ten wards in our study region, all in Kano state, which were visited between July 2015 and January 2016. Using the VTS database's boundaries as a starting point, we adjusted the boundaries for these ten wards, so that the boundaries matched as closely as possible the extent of the georeferenced eTally points, to prevent boundary discrepancies from influencing the validation results. The area covered by each residential type in each eTally ward in Kano was recorded in a portion table of the format shown in Table 2, so that the ward population could be simulated. The estimated under-5 population is the product of the simulated total population of the ward and the estimated under-5 fraction nearest the ward's centroid.

### 2.10 Bottom-up population mapping vs. census-based growth estimates

A major motivation for our work was the prevalence of postcensal population estimates that are made by applying constant growth rates to extrapolate outdated census counts, which can be problematic when true growth rates vary from place to place and from year to year, especially as the census date recedes further into the past. Without a more current full census, there is no way to know with certainty how large the errors are in such estimates, but our bottom-up approach, if conducted for two separate points in time, offers an alternative method for estimating growth rates. To demonstrate, we developed an historical settlement layer for the metropolitan area of Kano, which we used in conjunction with the population density model (Model 2) to make population estimates that approximately align temporally with the census. The historical settlement layer was based on Quickbird imagery from late 2005/early 2006, while the recent imagery for the Kano metropolitan area was collected in early 2014. Generating an output for 2006 to supplement our more current output allowed for comparison between our model and the census counts for 2006, as well as a comparison between the postcensal growth rates suggested by our methods and the constant growth rates commonly used. The metropolitan area consists of eight LGAs: Dala, Fagge, Gwale, Kano Municipal, Kumbotso, Nassarawa, Tarauni, and Ungogo.

## 3 Results

### 3.1 Settlement mapping

We used SMT to produce a settlement layer at approximately 7.5 m resolution, representing developed and undeveloped land across Kano and Kaduna. Table 3 shows that nearly all the land area of these states is undeveloped. Less than 3% of the land area of Kano and < 1% of the land area of Kaduna are covered by the settlement layer. The settlement layer is thus the single most important input for locating where the population resides in these states (by excluding the 98.54% of the land area where people do not live). The residential classifications and the associated population model have only to explain the variation in population density within the 1.46% of the area that is populated.

**Table 3:** Total area and settlement layer area by state.

State	Total area (km^2^)	Settlement area (km^2^)	% settlement
Kaduna	46,053	423.3	0.92%
Kano	20,131	541.7	2.69%
Total	66,184	965.0	1.46%

### 3.2 Population estimates

We produced gridded estimates of population counts across all of Kano and Kaduna, and generated prediction intervals for selected ROIs, including every ward in the two states, as well as the entire state of Kano. Because we implemented two distinct models of the residential population in Kano and Kaduna (one that leveraged the information in the residential classification (Model 2) and one that did not (Model 1)), we were able to assess the impact that the residential classification had on our ability to explain variations in population density. At high levels of aggregation (specifically the state level), we found that Models 1 and 2 provide similar point estimates, but that Model 2 provides narrower prediction intervals, owing to the explanatory power of the residential type classification. In the case of the state of Kano, we estimated (per Model 2) a total population of 13,688,669 in 2013 (the median year represented by the input satellite imagery). While the mean predictions between the two models were similar, Model 2's 90% prediction interval for the state was [12,517,841, 14,922,332], while the equivalent interval from Model 1 was [12,091,788, 15,041,447]. In other words, incorporating the residential types resulted in an 18% reduction (about 545,000) in the width of the prediction interval between the two models.

If we look at a finer spatial scale, specifically the ward level, patterns in uncertainty across space become clearer. Fig. 5 shows prediction interval widths for individual wards in the city centers of both Kano and Kaduna, alongside the settlement type layer for each city. Here, the effect of within-type variation becomes clear. Wards dominated by types with low within-type variation, such as *D* and *F*, allow for narrower prediction intervals than do wards with higher within-type variation, such as *A* and *B*. (Refer to Fig. 4 for distributions by type.)

### 3.3 Estimating and validating sub-populations

The validation of our under-5 estimates against the eTally data is summarized in Fig. 6, which shows that our estimates of the under-5 population are well correlated with the eTally counts, but that the estimates tend to be modestly lower than the eTally counts. Although the estimates fit very well with the reference counts (predictive *R*^2^ = 0.98), a zero-intercept linear regression suggests a slight underestimation bias, whereby a function of 0.942 times the reference data exhibits a stronger fit with the estimates (*R*^2^ > 0.99).

### 3.4 Bottom-up population mapping vs. census-based growth estimates

Our settlement mapping results showed that the total settlement area in the Kano metropolitan area increased by > 40% between 2006 and 2014 (the entire metropolitan area was covered by 2014 imagery). A comparison of the settlement layers is shown in Fig. 7, and the built-up portion of total metro area between 1986 and 2014 is shown in Table 4. The 2006 and 2014 values are from our analysis, while the others are drawn from a previous study (Ayila et al., 2014). Clearly, there was a substantial increase in the rate of development in Kano after 2006. The imputed compound annual growth rate of built-up area between 2000 and 2006 was 2.02%, whereas between 2006 and 2014, it was 4.37% per year. This acceleration can only be captured in a model that directly accounts for the additional settlement area. The acceleration would be missed in projections based merely on tabular projections of past trends, such as the 2015 projection shown in Table 4. (Ayila et al., 2014 appear to have made a mistake in the projection to 2015 by only simulating 5 years of growth between 2005 and 2015. Simulating the full 10 years would result in a projection of about 23.7% for 2015, still substantially lower than the 27.81% we observed for 2014.)

**Table 4:** Built-up (settlement) portion of Kano metropolitan area between 1986 and 2015.

Year	Pct. built-up	Source
1986	13.85%	Ayila et al. (2014)
2000	17.38%	Ayila et al. (2014)
2005	19.34%	Ayila et al. (2014)
2006	19.61%	This analysis
2014	27.81%	This analysis
2015	21.70%	Ayila et al. (2014); Projected

Because the built-up area increased at a rate of 4.37% per year between 2006 and 2014, a similar acceleration in the population growth rate would be expected as well, but constant growth rates are often applied for population estimates in these contexts. Table 5 shows the Model 2 population estimates alongside UN estimates (United Nations, Department of Economic and Social Affairs, Population Division, 2014) and the 2006 census count. The UN estimates are for the urban “agglomeration” of Kano, which is assumed to be roughly equivalent to the eight-LGA metropolitan area shown in Fig. 7. There is general agreement among the census, the UN and Model 2 for 2006; the census count and the UN estimate fall within the prediction interval albeit near the low end. By 2014, however, the Model 2 estimate is higher than the UN estimate by more than one million people, which demonstrates the hazard of applying a constant 2.1% growth rate to an area being built up at a 4.37% annual rate. In the bottom-up paradigm, we need not assume any particular growth rate, because we can instead measure the built-up area via remote sensing for any point in the past for which we have appropriate imagery, and we can apply the population density model across that area. The further removed from the last reliable census, the more important it is to apply remote sensing methods to this problem.

**Table 5:** Kano metropolitan area population counts/estimates by year (census, UN World Urbanization Prospects estimates, and Model 2 estimates).

Year	Census count	UN estimate	UN growth rate	Model 2 estimates (90% interval)		
				Lower	Mean	Upper
2006	2,826,307	2,957,573	–	2,823,909	3,223,453	3,678,770
2007	–	3,021,321	0.021	–	–	–
2008	–	3,086,534	0.021	–	–	–
2009	–	3,152,969	0.021	–	–	–
2010	–	3,220,929	0.021	–	–	–
2011	–	3,290,353	0.021	–	–	–
2012	–	3,361,373	0.021	–	–	–
2013	–	3,433,724	0.021	–	–	–
2014	–	3,507,632	0.021	3,886,128	4,466,607	5,134,557

## 4 Discussion

### 4.1 Representations of population estimates

We described two distinct ways to represent our modeled population estimates, 1) a standard gridded format and 2) prediction intervals that can be defined for any region of interest (ROI). While the gridded format is familiar, straightforward, and convenient for many tasks, it lacks the improvements in precision and interpretation offered by the prediction interval framework. Performing zonal analyses in GIS software for an ROI using the gridded estimates is easy, but it is imprecise compared to the simulation method, which uses the size and composition of the ROI measured from the precise vector boundaries of the input layers. The smaller an ROI is, the more important this precision becomes. More importantly, the simulation method provides a way to express uncertainty about spatial population estimates with unprecedented flexibility. The ability to estimate prediction intervals that are specific to the size and composition of one's region of interest is a novel and important development. One limitation is that this representation cannot be shared simply as a single raster layer, as can gridded estimates. It is instead a collection of input layers tied together with software, which can render prediction intervals on the fly. An important next step in this work will be to integrate this method into a user-friendly application that can satisfy queries for intervals nearly as quickly and easily as zonal queries are satisfied currently for gridded layers.

### 4.2 Sources of uncertainty

The simulated prediction intervals presented in this work incorporate the natural within-type variation in population density across Kano and Kaduna as well as the parameter uncertainty associated with fitting the models to the sample. However, there are other sources of uncertainty that would, if incorporated in this method and properly accounted for, ultimately result in wider intervals.

First, we are treating each draw in the simulations as independent, which misses any possible autocorrelation among densities of neighboring small areas. Properly accounting for the spatial autocorrelation, which would result in wider, more accurate intervals, should be a priority for future work on this topic. Second, we are not accounting for possible commission and omission errors in the settlement layer and misclassifications in the residential type layer. These errors are substantially reduced by our supervision of the classifications, both in providing labeled training sets for the models as well as in carefully verifying that the results are correct and making manual adjustments as needed. But there will always be some error present, and the current lack of true reference data against which to measure the probability of these spatial errors means it is infeasible at this time to explicitly incorporate the errors into the prediction intervals. However, as more independent validation data become available and are investigated, we are very likely to be pointed to instances of these problems (i.e., in any case where a validation data point falls in a very low-density portion of our modeled distributions, a spatial data problem is likely to be found), which will help us better understand them and perhaps help in suggesting ways to mitigate their effects in the future.

**Fig. 2:**
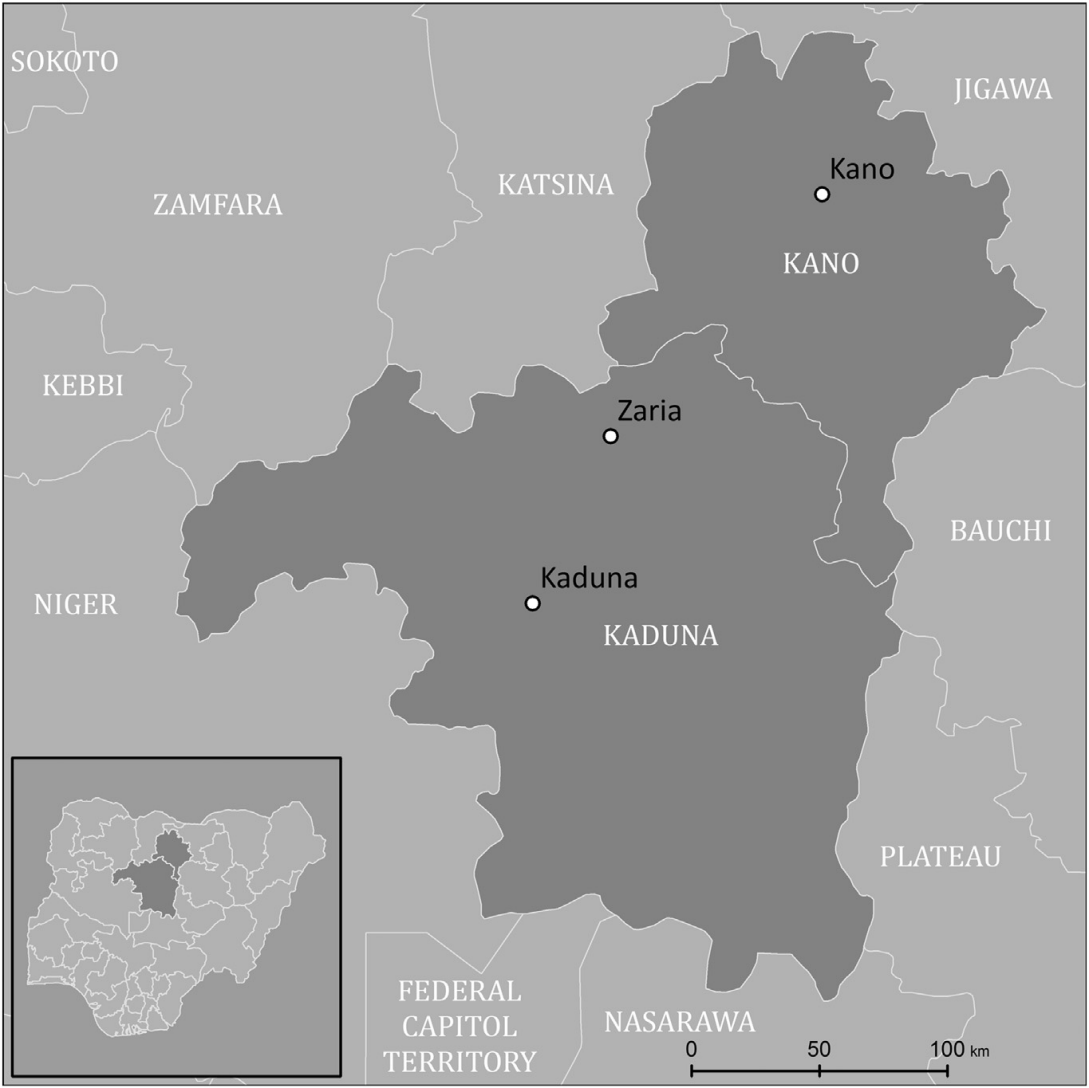
Kano and Kaduna states in Nigeria, with their three largest cities: Kano, Kaduna, and Zaria.

Aside from outright errors in the spatial data, there is also some variation in a variable that we call the *building area fraction* (BAF), which is the fraction of a given portion of the settlement layer that is actually covered by buildings. This variation arises from the fact that the settlement layer includes not just buildings, but also some surrounding non-built land. BAF variation originates in part in the variety of geometrical arrangements of human settlements (much of which is explained by the typology), but also in the variety of imagery and training data used. We propose that the best approach to mitigating the effects of BAF variation is to transition toward settlement mapping methods that more precisely outline buildings, with as little non-building area included as possible. For example, emerging building extraction methods using fully convolutional neural networks (Bittner et al., 2017, Maggiori et al., 2016, Yang et al., 2017) extract building area at the pixel level, such that in a well-trained model, BAF values are closer to one and less varied in comparison to coarser settlement mapping methods. In addition to reducing overall uncertainty and narrowing the population prediction intervals, reducing BAF variation could also eventually reduce the need for detailed residential classifications, given that building spacing is one of the primary components of the variation being captured by the residential typology (see Fig. 2).

**Fig. 3:**
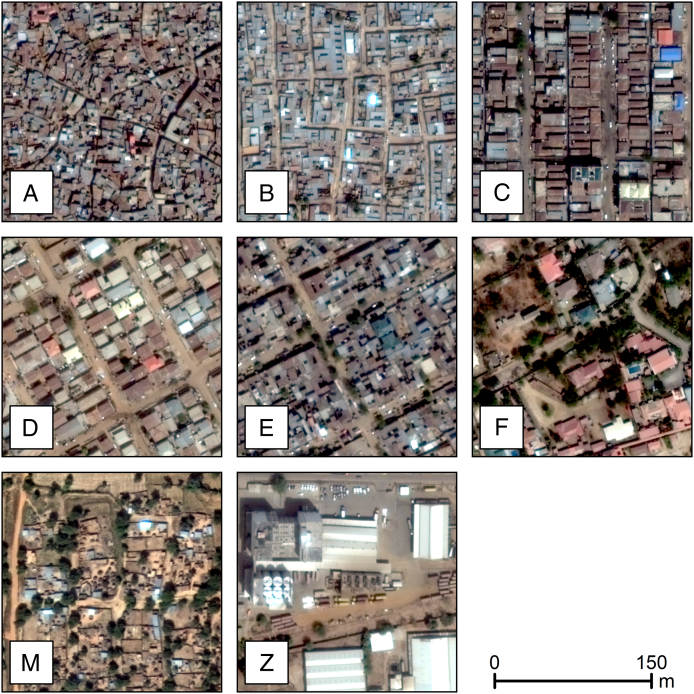
Exemplars of the urban residential (A–F), rural residential (M), and non-residential (Z) types for Kano and Kaduna states, Nigeria. The types vary in building size, building shape, building spacing, and formality/orthogonality of arrangement.

**Fig. 4:**
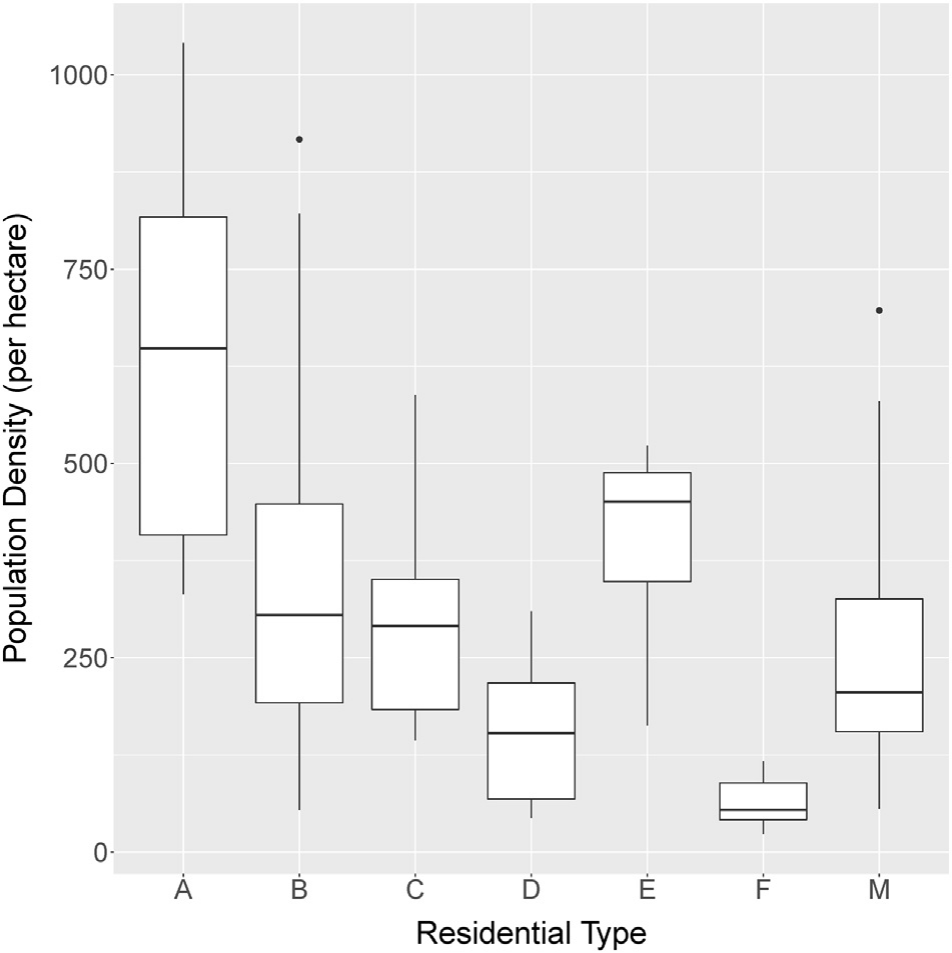
Population density by residential type.

**Fig. 5:**
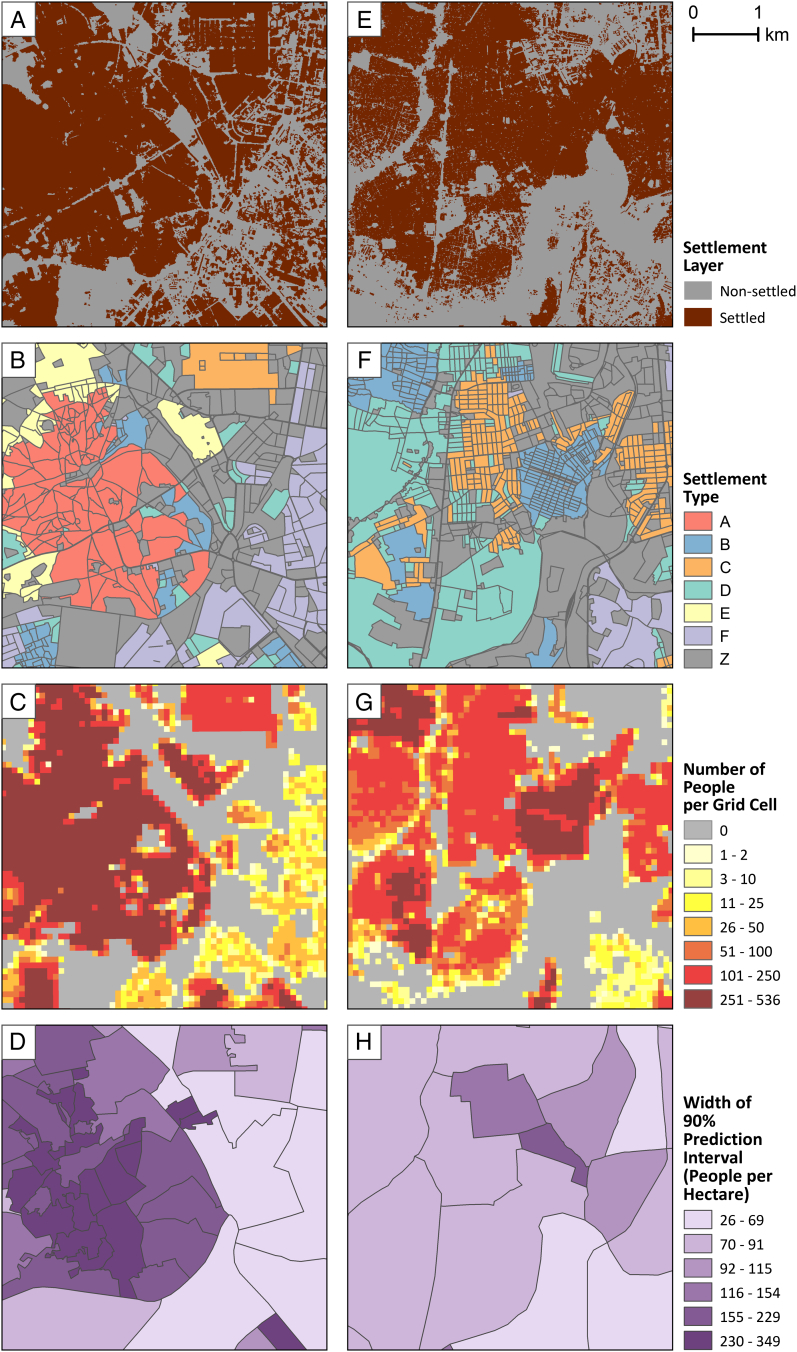
a) Settlement layer, b) settlement types, c) gridded population estimates, and d) 90% prediction intervals for administrative wards in the city center of Kano and e–h) the same layers in the city center of Kaduna. Intervals are in terms of population density (people per hectare).

**Fig. 6:**
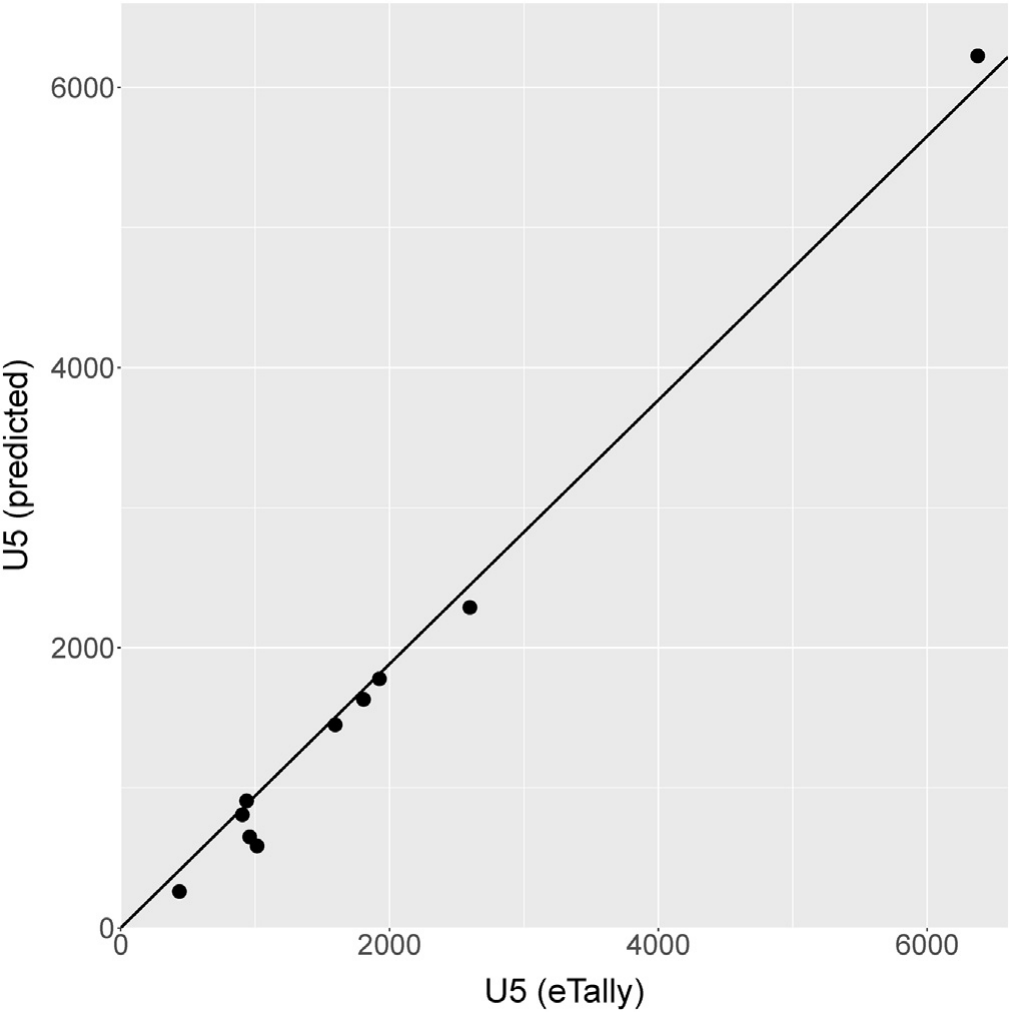
Under-5 (U5) population of ten Kano wards. Observed counts from the eTally program (see Section 2.9) are plotted against the model estimates. A zero-intercept regression line (*y* = 0.942*x*) is superimposed.

**Fig. 7:**
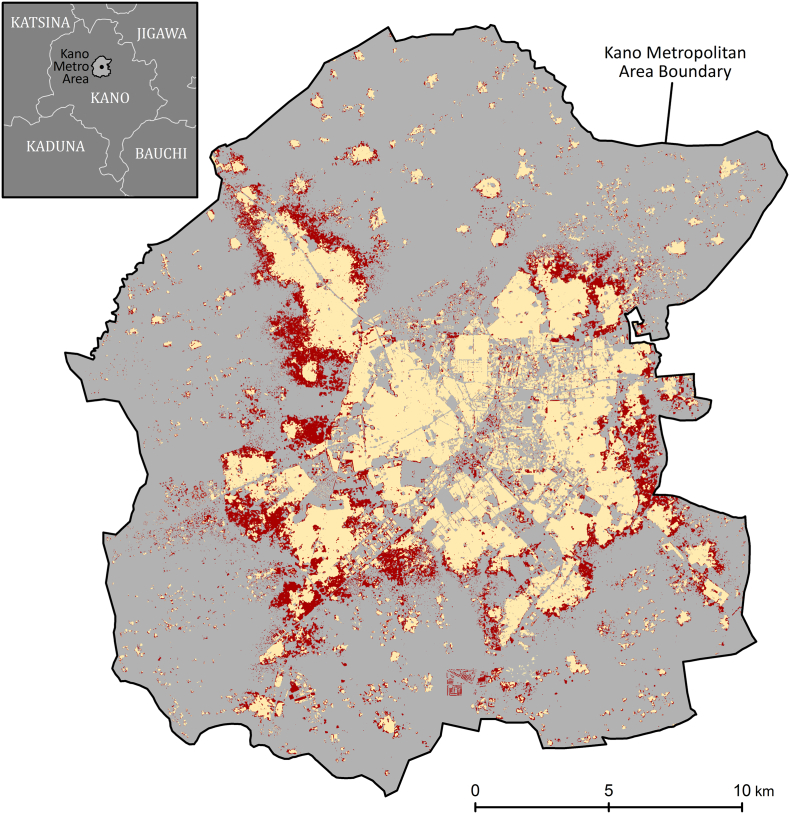
Urban growth in the Kano Metropolitan Area, 2006–2014. The settlement area detected from 2006 imagery is shown in tan and the additional settlement area detected in 2014 imagery is shown in red. (For interpretation of the references to color in this figure legend, the reader is referred to the web version of this article.)

Although the above concerns give us reason to suspect that our modeled intervals as currently implemented are probably too narrow to some degree, the current implementation allows for an expression of uncertainty not possible in the gridded representation of population as a single value at each cell in a raster, which is the most unrealistically narrow representation of all.

### 4.3 Model validation and assessment

Although our estimates of the under-5 population were very strongly correlated with the reference data, we did find a slight but consistent underestimation relative to the eTally counts. Because the population density model, the demographic model, and the eTally counts are derived from data collected using different survey methodologies, and because there is such a strong linear relationship between our predicted under-5 population and the eTally counts, it is likely that the explanation for the underestimations lies somewhere in the methodological differences among the three surveys. The contributing variables may include whether actual inhabitants (de facto) or usual inhabitants (*de jure*) were counted, how ages of respondents' children were ascertained and verified, and how non-compliant households were handled. These methodological questions cannot be treated adequately here, but a detailed exploration must be a priority for future work for further improving our estimation of subpopulations using disparate survey data. Another goal for future work should be to identify additional demographic datasets to allow for more extensive validations, beyond what was allowed by the ten-ward Kano eTally dataset used in this study.

Our population estimates for the Kano metropolitan LGAs in 2006 were close to the census-reported counts, though with an apparent modest overestimation with respect to the census. The census count does, however, fall within the 90% prediction interval. Any interpretation of this result must be accompanied by the caveats that the boundary of the metropolitan area/urban agglomeration is uncertain, that population densities within the settlement types may have been somewhat different in 2006, the imagery dates and census date are not perfectly aligned, and that the census counts themselves are uncertain. Therefore, rather than treating the census count as a gold standard against which we are assessing our model, the discrepancy between the census count and the model estimate should be thought of as a function of all of these uncertainties (boundary discrepancies, density changes, temporal alignment, census enumeration errors) as well as being a function of the microcensus sampling error. More important is our finding that the area of settlement of the metropolitan area increased at a rate of 4.37% per year after 2006, which explains why our approach yields a population estimate for Kano that is much higher than any published estimates, which project the population using growth rates that are much lower.

## 5 Conclusion

We have demonstrated a census-independent approach to making high-resolution population estimates using remote sensing methods and tailored microcensus surveys. Although there is inherent uncertainty when estimating population from only a sample enumeration, we introduced a method to quantify this uncertainty in the form of prediction intervals for any region of interest. We demonstrated the usefulness of residential classifications in explaining variability in population densities, the ability to estimate an important subpopulation, and the advantage that remote sensing methods can have over trend-based methods for postcensal estimation of populations for areas of rapid and accelerating growth. We do not expect or advocate widespread replacement of national censuses with the microcensus-based approach shown here, but we do recommend continued expansion of the use of surveys and settlement mapping for helping to quantify and understand the magnitude and characteristics of populations in areas where geospatial information is otherwise relatively scarce, inadequate, or outdated—which unfortunately includes much of the developing world.
